# Supplementary material for: Novel Genetic Diagnoses in Septo-Optic Dysplasia
Source: Genes (Basel). 2022 Jun 28;13(7):1165. doi: 10.3390/genes13071165 (PMC9320703; doi:10.3390/genes13071165)
Supplement: Supplementary file 1 [file genes-13-01165-s001.zip › genes-1780002-supplementary.pdf]

Supplemental Table S1. Variants of uncertain significance identified in Families 4-6.

Genomic coordinates	Ref	Alt	Gene	DNA change	Protein change	Segregation	gnomAd	CADD	REVEL
**Family 4 Quad analysis (affected brothers and unaffected parents)**									
Shared de novo ultra-rare coding variants									
None									
Shared compound heterozygous coding variants									
1:1550963	C	T	*MIB2*	NM_080875.3:c.-48C>T NM_001170688.1:c.124C>T	? p.(Arg42*)	Paternal (het)	4/185998	33	N/A
1:1563121	A	G	*MIB2*	NM_080875.3:c.1766A>G	p.(Gln589Arg)	Maternal (het)	270/259488	10.19	0.041
Shared homozygous coding variants									
None									
Shared X-linked coding variants									
X:49957529	C	T	*AKAP4*	NM_003886.3:c.1835G>A	p.(Cys612Tyr)	Maternal (het)	1/183267, 0 hemi	14.58	0.089
**Family 4 Shared heterozygous ultra-rare damaging coding variants**									
17:37343794	C	T	*CACNB1*	NM_000723.5:c.352G>A	p.(Glu118Lys)	Paternal (het)	NP	23.9	0.524
10:14050058	C	T	*FRMD4A*	NM_018027.5:c.46-149146G>A NM_001318336.2:c.93+1G>A	? Abnormal splicing	Paternal (het)	NP	24.1	N/A
12:118681285	G	A	*TAOK3*	NM_016281.4:c.229C>T	p.(Arg77*)	Paternal (het)	NP	37	N/A
3:100514690	G	A	*ABI3BP*	NM_001375547.2:c.3823C>T	p.(Gln1275*)	Paternal (het)	1/125478	18.08	N/A
20:43139995	T	C	*SERINC3*	NM_006811.4:c.410A>G	p.(Lys137Arg)	Paternal (het)	NP	30	0.619
1:6166503	C	T	*CHD5*	NM_015557.3:c.5809G>A	p.(Gly1937Arg)	Paternal (het)	NP	24.3	0.443
5:1065421	G	A	*SLC12A7*	NM_006598.3:c.2414C>T	p.(Pro805Leu)	Maternal (het)	2/228262	22.5	0.4
**Family 5 Trio analysis (affected proband and unaffected parents)**									
De novo ultra-rare coding variants									
None									
Compound heterozygous rare coding variants									
11:92565078	G	A	*FAT3*	NM_001008781.3:c.9772G>A	p.(Val3258Ile)	Paternal (het)	3/271900	24	0.131
11:92592376	G	A	*FAT3*	NM_001008781.3:c.11546G>A	p.(Arg3849Gln)	Maternal (het)	33/249054	24.2	0.41
Homozygous rare coding variants									
1:152129086	T	-	*RPTN*	NM_001122965.1:c.489delA	p.(Lys163Asnfs*48)	Maternal (het) Paternal (het)	NP	14.88	N/A
X-linked rare coding variants									
None									
**Family 5 Heterozygous ultra-rare damaging coding variants**									
1:226074655	A	T	*LEFTY1*	NM_020997.4:c.873T>A	p.(Tyr291*)	Maternal (het)	1/248920	35	N/A
6:84884666	A	T	*CEP162*	NM_014895.4:c.1805T>A	p.(Leu602*)	Maternal (het)	1/234868	34	N/A
15:63954095	CTGGCGGTTTGCA	-	*HERC1*	NM_003922.4:c.9015_9027delTGCAAACCGCCAG	p.(Ser3005Argfs*36)	Maternal (het)	NP	47	N/A
22:31494734	AG	-	*SMTN*	NM_134269.3:c.2243_2244delAG	p.(Glu748Alafs*21)	Paternal (het)	NP	43	N/A
17:38240169	A	T	*THRA*	NM_199334.5:c.304A>T	p.(Ile102Phe)	Paternal (het)	NP	23.9	0.725
9:2643628	C	G	*VLDLR*	NM_003383.5:c.821C>G	p.(Pro274Arg)	Maternal (het)	3/282824	26.4	0.598
9:73442865	G	A	*TRPM3*	NM_001366145.2:c.871C>T	p.(His291Tyr)	Paternal (het)	1/31412	23.2	0.419
**Family 6 Homozygous ultra-rare damaging coding variants**									
5:118582832	G	A	*DMXL1*	NM_005509.6:c.9002G>A	p.(Gly3001Glu)	N/A	1/251182	31	0.614
**Family 6 Heterozygous ultra-rare damaging coding variants**									

3:42788838	G	A	*CCDC13*	NM_144719.4:c.631C>T	p.(Gln211*)	N/A	NP	40	N/A
9:88938124	CTCTT	-	*TUT7*	NM_024617.4:c.2537_2541 delAAGAG	p.(Glu846Glyfs* 14)	N/A	NP	38	N/A
1:186143724	G	A	*HMCN1*	NM_031935.3:c.15893G>A	p.(Cys5298Tyr)	N/A	5/251202	29.7	0.968
2:227985864	C	T	*COL4A4*	NM_000092.5:c.193G>A	p.(Gly65Ser)	N/A	4/245712	27.8	0.927
3:151155750	A	C	*IGSF10*	NM_178822.5:c.6599T>G	p.(Ile2200Ser)	N/A	1/251388	26.5	0.906
15:66207856	C	A	*MEGF11*	NM_001385028.1:c.2425G> T	p.(Gly809Cys)	N/A	3/250960	31	0.904
10:71010337	G	A	*HKDC1*	NM_025130.4:c.1765G>A	p.(Gly589Ser)	N/A	1/251374	27.7	0.856
11:67800692	C	T	*NDUFS8*	NM_002496.4:c.314C>T	p.(Pro105Leu)	N/A	2/244040	25.1	0.748
6:4119433	A	G	*ECI2*	NM_206836.3:c.872T>C	,p.(Met291Thr)	N/A	NP	28.1	0.747
8:42608446	T	C	*CHRNA6*	NM_004198.3:c.1361A>G	p.(Asp454Gly)	N/A	3/237354	26.2	0.731
11:6472161	G	A	*TRIM3*	NM_033278.4:c.1831C>T	p.(Arg611Cys)	N/A	5/251348	32	0.68
12:51090928	C	T	*DIP2B*	NM_173602.3:c.2018C>T	p.(Ser673Phe)	N/A	NP	29.8	0.669
2:61192595	A	G	*PUS10*	NM_144709.4:c.640T>C	p.(Phe214Leu)	N/A	NP	25.1	0.649
1:155324278	C	G	*ASH1L*	NM_018489.3:c.7199G>C	p.(Gly2400Ala)	N/A	NP	28.9	0.514
1:55266778	T	C	*TTC22*	NM_001114108.2:c.59A>G	p.(Tyr20Cys)	N/A	NP	28.3	0.431
11:124766540	G	T	*ROBO4*	NM_019055.6:c.427C>A	p.(Pro143Thr)	N/A	NP	25.6	0.417
6:43014776	T	C	*CUL7*	NM_014780.5:c.2239A>G	p.(Asn747Asp)	N/A	2/251434	260	0.414

Hg19; gnomADv2.1.1; CADD Phred v1.4; N/A not applicable; NP not present
